# Nurses’ Silence Behavior as a Mediator Between Authentic Leadership and Near‐Miss Reporting Intention: A Cross‐Sectional Study

**DOI:** 10.1155/jonm/3998732

**Published:** 2026-06-22

**Authors:** In Hoe Ku, Sangjin Ko

**Affiliations:** ^1^ Department of Nursing, Ulsan University Hospital, Ulsan, South Korea, uuh.ulsan.kr; ^2^ Department of Nursing, University of Ulsan, Ulsan, South Korea, ulsan.ac.kr

**Keywords:** authentic leadership, near miss, patient safety, silence

## Abstract

**Background:**

Near‐miss reporting intention is essential for patient safety; however, reporting rates among nurses remain low. Nurses’ silence behavior—the intentional withholding of work‐related concerns—is a key barrier to reporting. While authentic leadership is known to promote nurses’ voice behavior, research examining the relationships between authentic leadership, nurses’ silence behavior, and near‐miss reporting intention remains limited. Therefore, this study aimed to examine the abovementioned relationships and to test the mediating role of nurses’ silence behavior.

**Methods:**

A predictive correlation design was used. Data were collected from 171 nurses at three general and tertiary hospitals in South Korea between August 20 and September 15, 2022. The mediating effect of silence behavior on the relationship between authentic leadership and near‐miss reporting intention was tested using the PROCESS macro (Model 4).

**Results:**

Authentic leadership was negatively associated with nurses’ silence behavior (*B* = −0.27, *p*  < 0.001), and silence behavior was negatively associated with near‐miss reporting intention (*B* = −0.65, *p* = 0.008). Authentic leadership did not have a significant direct effect on near‐miss reporting intention (*B* = 0.36, *p* = 0.142) but had a significant indirect effect through the mediator (*B* = 0.18, 95% CI = 0.03–0.37).

**Conclusion:**

Authentic leadership is indirectly linked to higher near‐miss reporting intentions through lower individual silence behavior among nurses. To enhance reporting intentions, it is necessary to mitigate situational constraints associated with silence and foster an environment conducive to open communication.

**Implications for Nursing Management:**

Nurse managers should consistently practice authentic leadership to dismantle rigid hierarchical atmospheres and cultivate an open communication climate. This targeted strategy may help reduce nurses’ silence behavior and encourage active participation in patient safety–strengthening behaviors, thereby creating a secure healthcare setting.

## 1. Introduction

Patient safety refers to the prevention of errors and adverse events that may occur during the delivery of healthcare services [1]. Among patient safety incidents, near misses refer to events that could have caused harm to the patient but were detected before any actual damage occurred [2, 3]. Near misses are regarded as precursors to medical errors and occur more frequently than actual medical errors, thus attracting considerable attention in patient safety efforts [4–6]. Encouraging near‐miss reporting allows for early detection and correction of errors before adverse events occur, providing a low‐cost learning opportunity [5, 7]. This process helps establish preventive measures and raises awareness of the causes of incidents, thereby improving patient safety management and healthcare quality [7, 8]. Nurses play a critical role in identifying the early signs of patient risk and responding to safety issues, making them well positioned to recognize near‐miss situations effectively [9, 10]. However, the rate of near‐miss reporting remains low [4, 5, 10]. Barriers to near‐miss reporting include limited awareness and knowledge regarding near misses [4], fear of reporting [4, 10], unclear reporting protocols and systems [4, 5, 8], insufficient error feedback [4, 5], insufficient preventive measures [5], and organizational culture, such as punishment and blame [8, 10]. Therefore, multifaceted approaches that consider both individual and organizational factors may be important in supporting nurses’ near‐miss reporting intentions.

Silence behavior among nurses refers to the intentional withholding of information, opinions, or concerns related to work situations [11, 12]. In healthcare settings, such silence behavior may prevent nurses from speaking up about safety‐related concerns and is negatively associated with patient safety outcomes [13–15]. Despite the potential harm to patient safety, nurses often choose to remain silent about perceived problems due to fear of negative consequences [8, 12]. These include interpersonal consequences, such as fear of retaliation [14] and isolation [12], as well as organizational consequences, such as being placed on probation, suspension, or even termination [8]. Additionally, reporting medical errors is often perceived as blaming colleagues, which further discourages nurses from speaking up [8]. Therefore, supervisory responsiveness and attitude are key contextual features in promoting error reporting among staff [8, 16]. Creating a positive organizational climate for error reporting is a crucial responsibility of nurse managers and is closely related to their leadership style [16]. Among leadership types, authentic leadership is characterized by high self‐awareness, an internalized moral perspective, balanced information processing, and transparency in relationships among members [17]. Authentic leadership fosters trust in managers, enhances work engagement [18, 19], and has been associated with increased unit care quality [19], reduced staff turnover [20], and, more recently, the promotion of a strong safety culture in healthcare settings [19, 21]. This leadership style has been shown to be a positive predictor of nurses’ voice behavior [18]. Voice behavior, in this context, includes the willingness and courage to identify problems and express them openly [18, 22]. This type of proactive communication plays a critical role in enhancing patient safety by promoting error reporting and encouraging nurses to actively participate in identifying ways to improve quality of care [18].

Previous studies [17, 18] demonstrated that leader transparency enhances member trust and facilitates reporting behavior, supporting the hypothesis that authentic leadership is directly associated with higher near‐miss reporting intentions. In addition, silence behavior is a major factor hindering near‐miss reporting intentions [14] and occurs primarily in environments with low psychological safety [10]. Considering previous studies [8, 23] showing that authentic leadership may reduce silence behavior by creating a safe and transparent atmosphere, authentic leadership is likely to have an indirect relationship with reporting intentions through its relationship with nurses’ silence behavior. Although this leadership style is expected to contribute both directly and indirectly to near‐miss reporting intentions, there is still a lack of research examining the relationships between these variables in nursing settings. Therefore, this study aimed to examine the relationships among authentic leadership, silence behavior, and near‐miss reporting intention and to test the mediating role of nurses’ silence behavior. Based on this framework, the following hypotheses are proposed. (H1) Authentic leadership is positively associated with near‐miss reporting intentions. (H2) Authentic leadership is negatively associated with silent behavior. (H3) Silence behavior is negatively associated with near‐miss reporting intentions. (H4) Silence behavior mediates the association between authentic leadership and near‐miss reporting intention.

## 2. Methods

### 2.1. Study Design and Participants

This study employed a predictive correlational design to examine the relationship between the variables. The participants were recruited using convenience sampling from three general and tertiary hospitals located in City U. After obtaining official permission from the nursing department of each hospital, recruitment notices were posted on the ward bulletin boards. The study included staff nurses directly involved in patient care, who understood the purpose of the study and voluntarily provided written informed consent. Nurses with less than 6 months of clinical experience were excluded to ensure that the study comprised participants who had sufficiently adapted to the organization and had accumulated professional nursing experience.

The minimum required sample size was initially calculated for multiple regression analysis using G∗Power, with a significance level (α) of 0.05, a power (1 − β) of 0.80, an effect size of 0.15, and 18 predictors, resulting in a minimum sample size of 150. A total of 180 nurses were recruited, with a potential dropout rate of approximately 20%. Of 180 questionnaires distributed, 175 were returned. After excluding four questionnaires due to incomplete responses or failure to meet the inclusion criteria, a final total of 171 responses were used for data analysis.

### 2.2. Instruments

A structured questionnaire consisting of general and near‐miss–related characteristics (12 items), authentic leadership (29 items), silence behavior (31 items), and near‐miss reporting intention (3 items) was used.

#### 2.2.1. General and Near‐Miss–Related Characteristics

The questionnaire for participants’ characteristics included general characteristics such as sex, age, marital status, education level, clinical experience, department, work shift type, position, and average daily working hours. Additionally, near‐miss–related characteristics included experiences with near‐miss events, awareness of near‐miss events, and knowledge of near‐miss reporting systems. Awareness was measured using a 5‐point Likert scale ranging from “*not at all*” (1 point) to “*very well*” (5 points), with higher scores indicating a higher level of awareness.

#### 2.2.2. Authentic Leadership

The Authentic Nurse Leadership Questionnaire (ANLQ) developed by Giordano‐Mulligan et al. [24] was used to measure the degree of authentic leadership of nurse managers as perceived by the participants. This instrument consists of five subdomains: self‐awareness (6 items), moral/ethical courage (4 items), relational integrity (4 items), shared decision‐making (6 items), and caring (6 items). Each item was rated on a 5‐point Likert scale ranging from 1 (“*strongly disagree*”) to 5 (“*strongly agree*”), with higher scores indicating higher levels of authentic leadership. The reliability measured by Cronbach’s alpha was 0.98 [24], and in the present study, Cronbach’s alpha was 0.96.

#### 2.2.3. Silence Behavior

To measure silence behavior, the Hospital Nurses Silence Behavior Scale (HNSBS), developed by Eriguc et al. [25] for Turkish nurses, was used. The scale consists of 31 items classified into two subfactors: situations leading to silence (16 items) and motivations for silence (15 items). Each item is rated on a 5‐point Likert scale ranging from 1 (“*never*”) to 5 (“*always*”), with higher scores indicating a greater degree of silence behavior among nurses. The scale demonstrated satisfactory reliability at development (Cronbach’s α = 0.96) [25] and in the present study (Cronbach’s α = 0.91).

#### 2.2.4. Near‐Miss Reporting Intention

Near‐miss reporting intention was measured using the intention to report near misses developed by Kim [26] for clinical nurses. This instrument consists of three subitems: intention to report my near miss, intention to report others near misses, and intention to share my near‐miss experience with others. Each item is rated on a numeric rating scale ranging from 0 (“*no intention to report*”) to 100 (“*very high intention to report*”), with higher scores indicating greater near‐miss reporting intention. The scale demonstrated satisfactory reliability at development (Cronbach’s α = 0.85) [26] and in the present study (Cronbach’s α = 0.88).

### 2.3. Data Collection

Data were collected from August 20 to September 15, 2022. Before data collection, the researcher visited the nursing departments and wards to explain the purpose and procedures of the study to nurse managers and obtained permission to proceed. The researcher personally delivered the questionnaires to each ward and provided detailed explanations to the nurses who met the inclusion criteria while also voluntarily agreeing to participate. The participants were assured that their responses would be kept confidential and used only for research purposes, with guaranteed anonymity. Written informed consent was obtained before completion of the questionnaire. To maintain privacy, the completed questionnaires were collected in sealed envelopes.

### 2.4. Ethical Considerations

This study was approved by the Institutional Review Board (IRB) of Ulsan University Hospital (Approval No. UUH 2022‐07‐013‐002). To ensure anonymity and confidentiality, personally identifiable information such as names, employee IDs, and department affiliations was not collected. The participants were provided with a small token of appreciation for their time. To prevent data leakage, all collected data were processed electronically and stored in a password‐protected external hard drive. The principal investigator managed all the aspects of data security. In accordance with the institutional data retention policies, the data will be securely stored for 3 years following the completion of the study for audit purposes, after which all records will be permanently and irreversibly destroyed.

### 2.5. Data Analysis

The collected data were analyzed using IBM SPSS Statistics Version 28.0 (IBM Corp., Armonk, NY, USA). Descriptive statistics, including frequencies, percentages, means, and standard deviations, were used to summarize the participants’ general characteristics and study variables. Pearson’s correlation analysis was conducted to examine the relationships between the variables. Data normality was evaluated using skewness and kurtosis values to assess the appropriateness of parametric statistical tests. The absolute values of skewness ranged from 0.06 to 0.79, and kurtosis ranged from 0.08 to 1.07, indicating that all variables satisfied the assumptions of normality.

Model 4 of the PROCESS macro for SPSS (v. 4.2) developed by Hayes [27] was used to examine the indirect association of authentic leadership with near‐miss reporting intentions through silence behavior. The indirect effect was evaluated using bootstrapping with 5000 resamples, and statistical significance was determined when the 95% confidence interval (CI) did not include zero. All statistical significance levels were set at *p* < 0.05.

## 3. Results

### 3.1. General and Near‐Miss–Related Characteristics of Participants

Of the participants, 93.0% were female. Most were in their 20s (51.5%), followed by those in their 30s (33.3%) and 40 or older (15.2%). Single nurses accounted for 60.8%, and most participants held a bachelor’s degree (75.4%). The largest group had more than 10 years of experience (33.9%). Most participants worked in general wards (60.2%), compared to 39.8% in other departments (intensive care units, outpatient clinics, emergency departments, operating rooms, dialysis centers, etc.). The majority worked three shifts (80.1%) and were staff nurses (86.5%). The average daily working hours were 8.41 ± 0.73 h.

Regarding near‐miss experience and awareness, 39.2% (*n* = 67) of the participants reported having experienced a near miss. The average awareness score for near misses was 3.40 ± 0.91, and the awareness of the near‐miss reporting system averaged 3.11 ± 0.89 points (Table 1).

**TABLE 1:** General and near‐miss–related characteristics of participants (*N* = 171).

Variables	Categories	*n* (%) or mean ± SD
Gender	Male	12 (7.0)
	Female	159 (93.0)

Age (years)	20 ∼ < 30	88 (51.5)
	30 ∼ < 40	57 (33.3)
	≥ 40	26 (15.2)

Marital status	Single	104 (60.8)
	Married	67 (39.2)

Education level	Diploma	39 (22.8)
	Bachelor	129 (75.4)
	≥ Master	3 (1.8)

Clinical experience (years)	< 3	32 (18.7)
	3 ∼ < 6	52 (30.4)
	6 ∼ < 10	29 (17.0)
	≥ 10	58 (33.9)

Department	Ward	103 (60.2)
	Others	68 (39.8)

Working shift type	Three shifts	137 (80.1)
	Non‐shift	34 (19.9)

Position	Staff nurse	148 (86.5)
	Charge nurse	23 (13.5)

Average daily working hours		8.41 ± 0.73

Experience of near‐miss events	Yes	67 (39.2)
	No	104 (60.8)

Awareness of near‐miss events		3.40 ± 0.91

Awareness of the near‐miss reporting system		3.11 ± 0.89

Abbreviation: SD, standard deviation.

### 3.2. Levels of Authentic Leadership, Silence Behavior, and Near‐Miss Reporting Intention

Perceived authentic leadership averaged 3.89 ± 0.74 points. Among its subdomains, self‐awareness scored the highest (3.98 ± 0.76 points), while moral ethical courage scored the lowest (3.81 ± 0.80 points). The silence behavior score was 88.04 ± 21.83 points (maximum 148). Specifically, within the silence behavior domains, the situation domain averaged 47.08 ± 11.73 points, and the motivation domain averaged 40.95 ± 12.66 points. The mean near‐miss reporting intention was 70.96 ± 22.56 points (maximum 100) (Table 2).

**TABLE 2:** Levels of authentic leadership, silence behavior, and near‐miss reporting intention (*N* = 171).

Variables	Categories	Mean ± SD	Min	Max
Authentic leadership	Total score	3.89 ± 0.74	1	5
	Self‐awareness	3.98 ± 0.76	1	5
	Moral ethical courage	3.81 ± 0.80	1	5
	Relational integrality	3.88 ± 0.79	1	5
	Shared decision making	3.90 ± 0.79	1	5
	Caring	3.83 ± 0.83	1	5

Silence behavior	Total score	88.04 ± 21.83	31	148
	Situation	47.08 ± 11.73	16	76
	Motivation	40.95 ± 12.66	15	75

Near‐miss reporting intention	Total score	70.96 ± 22.56	0	100
	Intention to report my near‐miss	71.93 ± 25.79	0	100
	Intention to report others’ near‐miss	64.80 ± 28.27	0	100
	Intention to share my near‐miss experience with others	76.14 ± 21.40	0	100

Abbreviation: SD, standard deviation.

### 3.3. Correlations Among Authentic Leadership, Silence Behavior, and Near‐Miss Reporting Intention

Near‐miss reporting intention was significantly positively correlated with authentic leadership (*r* = 0.17, *p* = 0.026), while showing a statistically significant negative correlation with silence behavior (*r* = −0.24, *p* = 0.002). Perceived authentic leadership was also negatively correlated with silence behavior (*r* = −0.27, *p* < 0.001) (Table 3).

**TABLE 3:** Correlations among authentic leadership, silence behavior, and near‐miss reporting intention (*N* = 171).

Variables	Authentic leadership	Silence behavior
	*r* (*p*)	
Silence behavior	−0.27 (< 0.001)	
Near‐miss reporting intention	0.17 (0.026)	−0.24 (0.002)

### 3.4. Mediating Role of Silence Behavior in the Association Between Authentic Leadership and Near‐Miss Reporting Intention

The mediating role of nurses’ silence behavior in the association between perceived authentic leadership and hospital nurses’ near‐miss reporting intention was analyzed using Model 4 of the PROCESS macro in SPSS.

**FIGURE 1:**
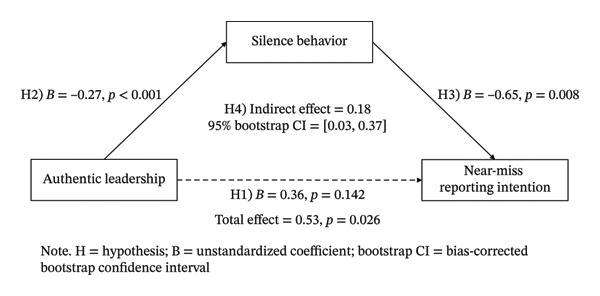
Mediating role of silence behavior in the association between authentic leadership and near‐miss reporting intention.

First, regarding the association between the independent and dependent variables, perceived authentic leadership did not have a significant direct association with near‐miss reporting intentions (*B* = 0.36, *p* = 0.142). Thus, Hypothesis 1 was not supported. However, the total relationship of authentic leadership with near‐miss reporting intention, which included both direct and indirect pathways, was statistically significant (*B* = 0.53, *p* = 0.026). Second, perceived authentic leadership was a significant negative predictor of nurses’ silence behavior (*B* = −0.27, *p* < 0.001), thereby supporting Hypothesis 2. Third, nurses’ silence behavior was found to be a significant negative predictor of near‐miss reporting intention (*B* = −0.65, *p* = 0.008), supporting Hypothesis 3. Finally, to test the mediating role of nurses’ silence behavior (Hypothesis 4), a bootstrapping analysis with 5000 resamples was conducted. The indirect effect of authentic leadership on near‐miss reporting intention through silence behavior was 0.18, with a 95% bias‐corrected CI of 0.03–0.37, excluding zero. These results confirm that silence behavior significantly mediates the association between authentic leadership and near‐miss reporting intention, supporting Hypothesis 4. As no significant direct effect was observed, the relationship between authentic leadership and near‐miss reporting intention appears to be primarily explained by an indirect pathway through silence behavior (Table 4, Figure 1).

**TABLE 4:** Mediating role of silence behavior in the association between authentic leadership and near‐miss reporting intention (*N* = 171).

Pathway	*B*	SE	β	*t* (*p*)	*R* ^2^	*F* (*p*)	95% CI	
							LLCI	ULCI
Direct path: authentic leadership ⟶ silence behavior	−0.27	0.08	−0.27	−3.62 (< 0.001)	0.07	13.07 (< 0.001)	−0.42	−0.12
Direct path: silence behavior ⟶ near‐miss reporting intention	−0.65	0.24	−0.21	−2.70 (0.008)	0.07	6.24 (0.002)	−1.12	−0.17
Direct path: authentic leadership ⟶ near‐miss reporting intention	0.36	0.24	0.11	1.47 (0.142)	0.07	6.24 (0.002)	−0.12	0.84
Total path: authentic leadership ⟶ near‐miss reporting intention	0.53	0.24	0.17	2.24 (0.026)	0.03	5.01 (0.026)	0.06	1.01
Indirect effect: authentic leadership ⟶ silence behavior ⟶ near‐miss reporting intention	0.18	0.09					0.03	0.37

*Note:* B = unstandardized coefficient; β = standardized coefficient.

Abbreviations: LLCI = lower‐level confidence interval, SE = standard error, ULCI = upper‐level confidence interval.

## 4. Discussion

The results showed that perceived authentic leadership was significantly and negatively associated with nurses’ silence behavior, and this silence behavior was further linked to lower near‐miss reporting intention. Furthermore, nurses’ silence behavior was identified as a significant mediating factor in the association between authentic leadership and near‐miss reporting intention. These findings are supported by previous studies demonstrating that nursing leadership styles significantly shape the workplace climate [8, 23] and that nurses working in environments with higher psychological safety exhibit a greater willingness to participate in safety‐enhancing behaviors such as error reporting [8, 14, 28]. Interestingly, the results showed that authentic leadership did not have a direct significant association with near‐miss reporting intention; rather, the relationship was primarily explained by an indirect pathway through nurses’ silence behavior. This suggests that even if a manager demonstrates high authentic leadership, its positive association with near‐miss reporting intentions may be limited if nurses’ individual tendencies to remain silent are prominently manifested. It is particularly noteworthy that the scores for the “situational” dimension of nurses’ silence behavior were higher than those for the “motivation” dimension. This indicates that nurses’ silence is more closely linked to perceived situational constraints, such as a hierarchical workplace culture or an unfavorable climate for speaking up, rather than by individual psychological motivations. In healthcare settings, silence conceals warning signs of patient safety risks, thus hindering fundamental problem solving and posing a serious threat to patient safety systems [12, 29, 30]. Concerns about maintaining peer relationships or conforming to organizational norms may inhibit error reporting [29]. Therefore, priority must be given to strategies that mitigate these situational constraints and establish psychological safety to ensure that the reporting of patient safety incidents is perceived as acceptable and encouraged.

In today’s complex healthcare environment, authentic leadership is increasingly being recognized as an important approach in nursing practice [19, 31, 32]. Authentic leaders foster trust and transparency within nursing teams and help create conditions in which nurses feel more comfortable expressing concerns related to patient safety [17, 18, 21]. In this study, authentic leadership was associated with reduced silence behavior, which in turn was related to higher near‐miss reporting intentions. These findings suggest that lower nurses’ silence behavior may be an important mechanism through which leadership relates to patient safety–related behaviors.

Overall, the findings suggest that improving near‐miss reporting requires more than simply strengthening the reporting systems. Nurse managers should pay attention to contextual factors that discourage nurses from speaking up and foster a work environment in which safety‐related concerns can be communicated without hesitation. In this regard, authentic leadership may serve as an important leadership competency that supports near‐miss reporting by being associated with lower nurses’ silence behaviors and encouraging active participation in patient safety–promoting behaviors.

## 5. Conclusions

The results of this study show that authentic leadership is indirectly associated with near‐miss reporting intentions through silence behavior. To enhance reporting intentions, nurse managers should demonstrate authentic leadership and implement effective strategies to reduce nurses’ silence behavior, while simultaneously supporting an environment conducive to open communication. In addition, hospitals should promote positive workplace climate change and develop systematic education and reporting facilitation programs for ward nurses regarding near‐miss events.

This study had some limitations. First, the use of a correlational design limits the ability to infer causal relationships among the variables. Therefore, the identified mediating effects should be interpreted with caution. Second, convenience sampling was used to recruit 171 nurses. Therefore, caution should be exercised when generalizing the findings. Third, although the sample size met the requirements for a multiple regression analysis, mediation analyses generally benefit from larger samples to obtain more stable indirect effect estimates. Further studies with larger and more representative samples are needed to confirm these findings. Nevertheless, this study contributes to understanding the mechanism through which authentic leadership is associated with nurses’ patient‐safety behaviors through its relationship with silence behavior.

## 6. Implications for Nursing Management

To enhance patient safety, nurse managers should consistently demonstrate authentic leadership in their daily clinical practice. Transparent communication and supportive responses to safety‐related concerns may increase nurses’ willingness to report near miss events. Management strategies should, therefore, focus on reducing barriers that discourage speaking up and implementing leadership development programs that strengthen authentic leadership competencies. These efforts may contribute to safer and more communicative healthcare environments.

## Author Contributions

In Hoe Ku: conceptualization, investigation, data curation, formal analysis, writing–original draft preparation, and writing–reviewing and editing; Sangjin Ko: conceptualization, methodology, supervision, formal analysis, writing–original draft preparation, writing–reviewing and editing, and project administration.

## Funding

This research did not receive any specific grant from funding agencies in the public, commercial, or not‐for‐profit sectors.

## Disclosure

This manuscript is based on a part of the first author’s master dissertation from the University of Ulsan.

## Ethics Statement

This study was approved by the Institutional Review Board (IRB) of Ulsan University Hospital (Approval No. UUH 2022‐07‐013‐002). All data were coded to ensure anonymity. Participants were informed that participation was voluntary, that they could withdraw at any time without penalty, and that their data would be used solely for research purposes. Data were stored in a locked cabinet accessible only to the principal investigator and were destroyed after 3 years, in accordance with the Personal Information Protection Act.

## Conflicts of Interest

The authors declare no conflicts of interest.

## Data Availability

Data are available on request due to privacy/ethical restrictions.
